# Cork and Its Uses

**Published:** 1865-08

**Authors:** John R. Jackson


### SELECTED.

### CORK AND ITS USES.

By JOHN R. JACKSON.

Amongst the many materials or productions in use in every-day life, cork may certainly take a position in the foremost rank. We all know something of cork; from our earliest childhood we have been familiar with it. It is a substance that has retained all its ancient uses, as well as its importance and value, from its earliest history down to our own day. Unlike most other things, it has not, even in this age of application and invention, found a rival. True it is we have "corky" substances in abundance, produced in almost every country; but neither the productions of nature nor the productions of mechanical skill have produced an efficient substitute for cork, one that could take the place of this valuable bark, or even go side by side with it.

Considering the great quantity of cork that is consumed even in this country alone, as well as the great amount that is wasted, the quantity of bark annually stripped in the cork-forests is an operation of no little importance. The slight value many individuals place upon cork, on the whole, does not lead us in the least degree to estimate its real importance, which, in a commercial point of view, is of no trifling nature.

There must needs be a large quantity imported; for amongst wine merchants, bottled-beer merchants, or soda-water makers, a cork is never used a second time; but then what an immense bulk would go to make up a ton of cork, and yet it is by weight that the imports are estimated. There is an immense consumption, and the demand of late years has almost exceeded the supply. The annual quantity imported into this country averages about 5000 tons.

Of the early history of cork, it is very clear that it was well known and in use amongst the Greeks and Romans. Theophrastus distinctly alludes to the fact, now so well known, that the continual barking of the trees tends to improve the quality of the cork. With the Greeks it was called "Phenos," while the Romans knew it by its present specific name of "Suber." Though cork was probably used in very remote times for similar purposes to those of the present day—that of stoppers for bottles amongst the rest—this, however, does not seem to have been its common or general use, inasmuch as we find that vessels of that period were frequently closed by earth, clay, and other similar substances. Stoppers of cork, or "corks," as we now call them, appear not to have been generally introduced till some time in the latter part of the sixteenth century; from that period, however, its use has been getting more and more universal in all parts of the world.

Before the introduction of cork, or its general adoption for bottle-stoppers, various articles were resorted to for this purpose. We are told that apothecaries secured the contents of their phials with stoppers made of wax, which must have been a somewhat tedious process. But even in our own day, a similar custom prevails in many parts of Europe; for with many of the Italians and Neapolitans, for instance, the practice of securing their wines, by pouring oil into the mouth of the bottle before tying it down with skin, is still very prevalent.

Before entering into the uses of cork, however, let us pay a short visit to the forests from whence it is obtained, and trace its progress from its natural position to that of its ultimate application.

Cork, as we all know, is the bark of a tree, though commercially miscalled "corkwood." It is produced by two species of oak, *Quercus suber*, L., and *Quercus occidentalis*, hence called the "cork-oaks." These trees grow abundantly in large forests in Spain, Italy, the South of France, and Northern Africa, the latter species being found alone on the Atlantic side. This species is also peculiar, from the fact that it ripens its acorns in the second year.

In general appearance the cork-oaks differ little from the common oak, except, perhaps, that they do not attain to so large a size. There is also a slight difference in the form of their leaves—those of *Quercus suber*, L., being more lanceolate, and the margins not so deeply sinuate; the acorns are also somewhat longer and more tapering in form than those of the common oak.

The cork-oak does not require a rich soil; but, on the contrary, it seems to thrive best in poor and uncultivated ground.

To collect this cork, incisions are made longitudinally and transversely in the bark of the living tree, the instrument used being a kind of axe, the handle of which terminates in a wedge-shaped form. After the bark is cut through, it is beaten to loosen it from the liber or inner bark, the wedge-shaped axe-handle being inserted to lift the bark from the trunk. The cork thus removed usually varies from three-quarters of an inch to three inches in thickness. The next operation is to divide it into pieces of a uniform or convinient size, and to flatten it, each piece having, of course, a similar curve, corresponding with the trunk of the tree from whence it was taken. For this purpose, the pieces are placed in pits and covered with water, and then pressed flat with heavy stones. The well known charred surface upon these cork slabs is caused by the application of heat at an open fire, after the steeping, for the purpose of contracting the pores. The pieces are afterwards bound up in bales, in which form they appear in the market. In removing the cork from its paternal trunk, care has to be taken not to injure the inner bark next the wood, else it would affect the second crop of bark, and perhaps injure the tree. This operation of stripping the bark, if dexterously and carefully performed, has, as we have already said, no detrimental effect, either upon the growth of the tree or the rapid formation of the new bark; but, on the contrary, the tree is said to grow more hardy and vigorously. The first crop of bark is usually taken when the tree is about twenty-five or thirty years old, but the crop is of less value than that of any succeeding gathering, as it is harder, very uneven, and more full of holes. The second gathering, however, which is in about eight or ten years after the first, is still of an inferior quality. The third crop, collected in about eight years after the second, is usually the first marketable cork—that is, the first crop that is fit for cutting into bottle corks. When the trees have attained to this age, so that three crops have been taken off, they usually yield a supply of good cork about every seven or eight years; and its quality improves, as well as the quantity enlarging, at each successive gathering. The season chosen for the cork harvest is usually the month of July or August.

It will be seen by the foregoing that the quality, and consequently the commercial value of cork is materially affected by soil, length of time allowed in growing, and also of care in collecting. There is as much difference existing in the quality of cork as in most other articles of daily use. The finest kind should be compact and firm, but at the same time not hard, of an even texture or grain, and of a slightly pinkish tint. This kind of cork is generally selected by wine merchants for bottle-corks; while the coarser kind, which is always more porous, full of small holes, and perhaps punctured by insects, serves for bungs for casks and for the various other applications to which cork is put in a cheap form. When cork is required to be thick, it is usually found coarse, as it must be allowed a longer period of growth to promote its thickness. The charring or singeing process to which this kind of bark is frequently subjected, for the purpose of filling up the pores and making it impervious to fluids, has also a detrimental effect, as it secretes an empyreumatic oil, which is given off and frequently taken up by the liquids it confines; but there is no doubt that care is taken in the selection of these corks, and methods adopted for the prevention of this chemical contamination, as much as possible. This operation of charring, to which all cork was formerly subjected for the purposes we have just mentioned, has been partially succeeded of late by that of boiling the cork and afterwards scraping the surface. This is said to improve rather than to deteriorate the cork, it being more effectual in filling up the pores.

The uses of cork are so numerous, and its application so continually increasing, that the supply of late, as we have said before, has not been sufficient to meet the demand. It is not our intention to enumerate all the uses to which this most useful article is put—indeed, it would be unnecessary to do so, so well known as they are to all; but there are a few modern uses or applications to which cork has been found suited in recent inventions, and which are, perhaps, among the "things not generally known;" but these uses generally consume waste or refuse cork, such cuttings as were formerly considered of no value.

The new elastic floor-cloth, now so well known as "Kamptulicon," is a combination of caoutchouc and cork; and this is but one instance showing that cork, treated with other substances, can be made into a really useful article. Cork-dust has been used successfully with india-rubber in the process of vulcanizing, and to so fine a powder is it reduced for this purpose, that india-rubber so treated is capable of being moulded into the most delicate forms. Another recent application of cork is for stuffing beds, and we believe this is now done to a large extent.

A large Cork Company, lately established in London, and owning large forests in Portugal, have recently imported the virgin cork into this country, with the impression of its becoming useful for rustic garden-work. It is brought in very large pieces, and, from its rugged, uneven surface, which is frequently covered with lichens, together with its portability and its porous nature, which makes it capable of retaining moisture, will no doubt cause it to be much used for such purposes.

Though the bark of the cork-tree contains a considerable amount of tannin, it is not in general favor among tanners, on account of its not imparting the required "bloom;" and for this reason it is seldom used alone, but is mixed with English oak bark. The inner bark is that which is used for tanning purposes, the outer bark being quite devoid of any of the required properties. The removal of the inner bark causes the death of the tree; and it is chiefly from Sardinia and some parts of Spain, where the trees are very abundant, that it is imported for this purpose. The quantity of tannin, as well as the color of the bark, varies much, according to the district from whence it is obtained. The Sardinian bark is thicker and of a deeper red color than any other.

To return to cork itself and its more common applications, we find that there are two sorts or qualities known in commerce, called respectively white and black cork. The white, which is chiefly produced in the south of France, is the best, as it is smoother, of a more even and finer grain, and freer from holes and knots.

The operation of cork-cutting is one requiring great dexterity and neatness, and is carried on to a great extent both in France and England, though, as might be supposed, the French surpass, the English in this art. Machinery has been tried for the purpose of cork cutting, but all is now cut by hand. Considering the difficulty, with which we are all acquainted, of cutting a clean surface to cork, it is surprising to see the rapidity with which the workman turns out a perfect cork stopper from the little square pieces furnished to him. The knife used for this purpose has necessarily to be very sharp, as well as being very thin; the blade is broad, and when the edge has become dull, it is quickly sharpened on a very fine-grained stone. The bench or tube at which the workman sits has a ledge round it to prevent the corks falling off. On the Continent, a notch is made in the edge of the bench to place the back of the knife in, to prevent it from slipping. Thus the edge is uppermost, and the knife has to be guided slightly while the cork is pressed against the edge, and so dexterously turned and rounded to the required form. All the corks thus cut are thrown into a basket to be sorted, which is usually done by women and boys.

The great importance of cork as a commercial article has been the cause of experiments being tried for its introduction into the Southern States of North America. It is, however, some years since the American Government tried this plan of naturalization, for which purpose large quantities of the acorns were imported from the south of Europe. More recently, we learn, from Sir J. W. Hooker's last Report on the Royal Gardens, Kew, that steps are now being taken by the Colonial Government of South Australia to introduce the cork tree, and a number of young plants have been raised at Kew expressly for transmission to that colony.

We sincerely hope that these efforts to establish a tree furnishing so useful a product as cork, in a colony where it would become a valuable addition to its commerce, as well as adding to the supply, which, at the present increasing rate of consumption, is much to be desired, may be crowned with success.—*American Journal of Pharmacy*, from *The Technologist.*

